# Reporter Transgenes for Monitoring the Antitumor Efficacy of Recombinant Oncolytic Viruses

**DOI:** 10.32607/actanaturae.11719

**Published:** 2022

**Authors:** A. V. Semenova, G. F. Sivolobova, A. A. Grazhdantseva, A. P. Agafonov, G. V. Kochneva

**Affiliations:** Federal Budgetary Research Institution «State Research Center of Virology and Biotechnology «Vector», Koltsovo, Novosibirsk region, 630559, Russia

**Keywords:** oncolytic viruses, reporter transgenes, optical imaging, tumor cell, deep-tissue imaging, NIS

## Abstract

Accurate measurement of tumor size and margins is crucial for successful oncotherapy. In the last decade, non-invasive imaging modalities, including optical imaging using non-radioactive substrates, deep-tissue imaging with radioactive substrates, and magnetic resonance imaging have been developed. Reporter genes play the most important role among visualization tools; their expression in tumors and metastases makes it possible to track changes in the tumor growth and gauge therapy effectiveness. Oncolytic viruses are often chosen as a vector for delivering reporter genes into tumor cells, since oncolytic viruses are tumor-specific, meaning that they infect and lyse tumor cells without damaging normal cells. The choice of reporter transgenes for genetic modification of oncolytic viruses depends on the study objectives and imaging methods used. Optical imaging techniques are suitable for in vitro studies and small animal models, while deep-tissue imaging techniques are used to evaluate virotherapy in large animals and humans. For optical imaging, transgenes of fluorescent proteins, luciferases, and tyrosinases are used; for deep-tissue imaging, the most promising transgene is the sodium/iodide symporter (NIS), which ensures an accumulation of radioactive isotopes in virus-infected tumor cells. Currently, NIS is the only reporter transgene that has been shown to be effective in monitoring tumor virotherapy not only in preclinical but also in clinical studies.

## INTRODUCTION

To date, the use of oncolytic viruses is one of the most promising areas in cancer therapy. A great advantage of oncolytic virotherapy is that oncolytic viruses can specifically target tumor cells and lyse them without damaging healthy tissue during the entire treatment course [1]. Oncolytic viruses surpass the heterogeneity of tumor cells; they can lyse tumor stem cells, which are practically not amenable to other types of oncotherapy [2]. On the contrary, similar to the disruption of interferon signaling pathways in tumor cells, the immunosuppressive tumor microenvironment, which hinders effective immunotherapy, promotes virus replication [3]. At the same time, successful virus replication in the tumor inevitably makes the tumor more immunogenic due to pathogen-associated danger signals sent by infected cells (PAMP and DAMP). During tumor cell lysis, tumor-associated neoantigens are also released and an adaptive T-cell response to these antigens is formed [4]. Oncolytic viruses can act synergistically with other anticancer drugs, in particular, with checkpoint inhibitors (ipilimumab, atezolizumab, nivolumab, etc.) and CAR T-cell therapy [1, 5, 6].

Both natural and genetically modified virus strains of different taxonomic groups have oncolytic properties. The transgene insertion makes it possible to alter the properties of oncolytic viruses in a targeted manner, thus enhancing their tumor-specificity [7], ability to proliferate inside a tumor [8], as well as their immunostimulatory [9 , 10, 11] and cytolytic activities [12, 13]. Reporter transgenes, which can be used for non-invasive instrumental monitoring of the antitumor and antimetastatic activities of the virus, as well as its safety for other body organs and tissues, occupy a special place in the genetic modification of viruses [14, 15].

Non-invasive imaging studies are of great importance in the diagnosis and management of cancer patients [16]. The effectiveness of antitumor therapy directly depends on a timely and accurate diagnosis of tumor nodes and metastases, and monitoring of tumor response to therapy can help in selecting the optimal treatment strategy. Since reporter transgene expression is associated with viral replication, imaging can be used in preclinical and clinical studies as an early indicator of the therapeutic effect of oncolytic viruses [17]. Non-invasive imaging of the whole body of an experimental animal at a number of time points will help evaluate the efficiency of virus delivery to tissues of interest and allow monitoring and quantifying infection and expression of therapeutic transgenes during the treatment course.

**Fig. 1:**
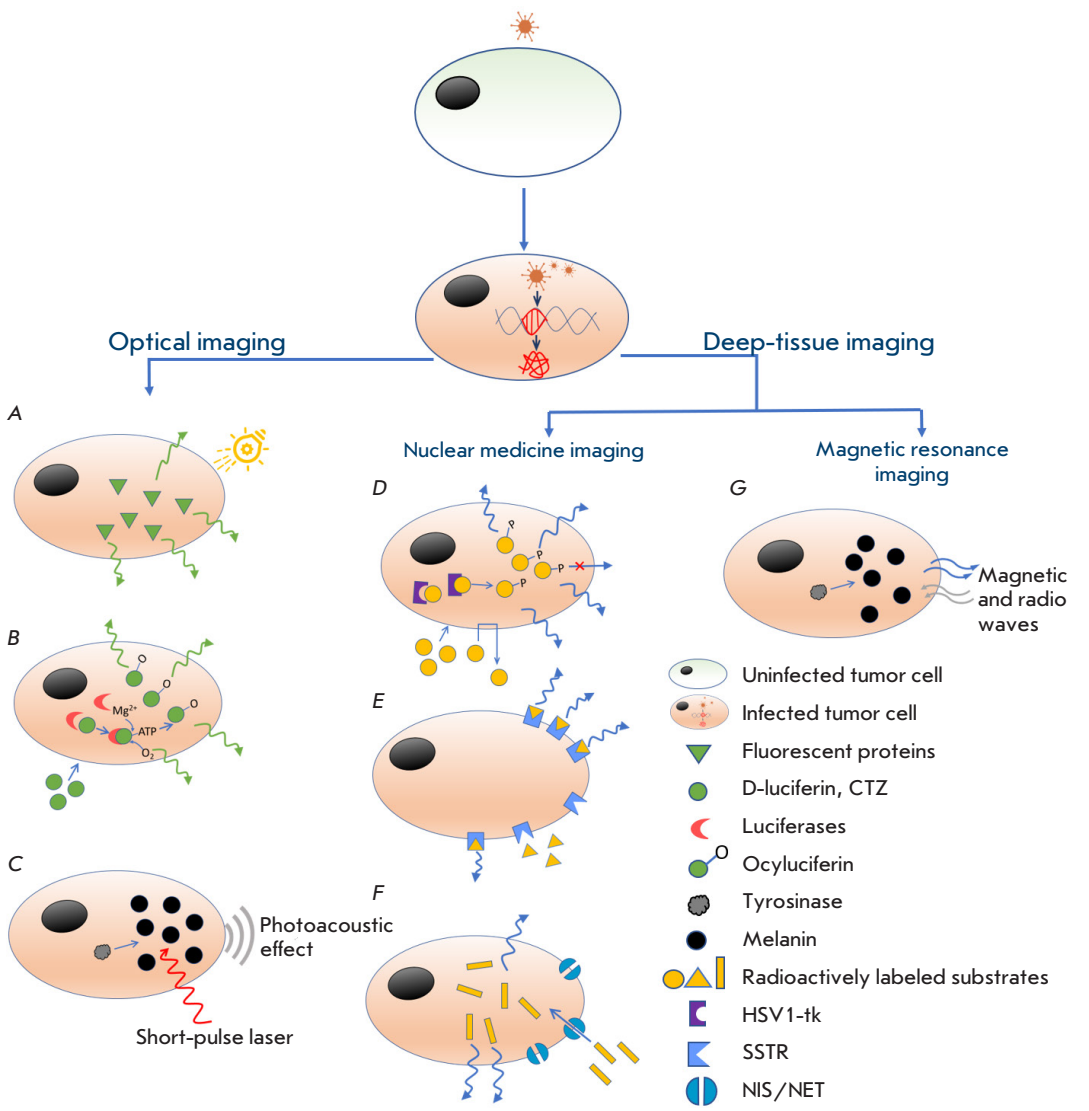
Tumor cell imaging methods using oncolytic viruses expressing protein reporter transgenes. Optical imaging methods: (*A*) – fluorescence imaging: fluorescent proteins emit fluorescence when irradiated with light of a certain wavelength; (*B*) – bioluminescence imaging: light is produced when exogenous substrates are oxidized by a bioluminescent reporter enzyme; (*C*) – photoacoustic imaging: a photoacoustic effect is achieved by irradiating a target tissue with a short-pulsed laser. Deep-tissue imaging techniques (*D*–*F*) – SPECT, PET; (*G*) – MRI; (*D*) – the HSV1-tk enzyme interacts with radioactively labeled substrates and converts them into a metabolite incapable of leaving the cell; (*E*) – the SSTR2 receptor binds radioactively labeled synthetic peptide substrates; (*F*) – NIS/NET transporters ensure the absorption and accumulation of radioactive substrates inside the cell; (*G*) – melanin enhances the magnetic resonance signal

Imaging techniques can be divided into the following categories: optical imaging using non-radioactive substrates, deep-tissue imaging using radioactive substrates, and magnetic resonance imaging (Fig. 1). In the following sections, we will focus on the most studied reporter transgenes from oncolytic viruses used in various non-invasive imaging techniques.

## OPTICAL IMAGING

Optical imaging is based on the use of light in the infrared, visible, and ultraviolet spectra. For this reason, it is best suited for imaging of surface than deep tissues. Optical imaging techniques used to detect reporter transgenes from oncolytic viruses include fluorescence, bioluminescence, and photoacoustic imaging (Fig. 1). The depth of the possible imaging of optical techniques varies over a fairly wide range: from fractions of a millimeter to several centimeters, and depends both on the chosen technique and absorption/ scattering of excitation light and/or light emitted by surrounding tissues.

**Fluorescence imaging**

Fluorescence requires incident light of appropriate excitation wavelength to reach the fluorophore, causing the fluorophore to emit a photon of a specific wavelength. The emitted photons are detected using a highly sensitive CCD camera installed in a light-tight box.

Genes encoding such fluorescent proteins as the green fluorescent protein (GFP) and its enhanced variants, red fluorescent protein (RFP), yellow fluorescent protein (YFP), mCherry, and many others, are used as reporter genes for fluorescence imaging [18, 19].

GFP, first isolated from the Aequorea victoria jellyfish in 1960 [20], quickly became one of the most widely used and studied proteins in biochemistry and cell biology [21]. The protein is used because of its ability to generate a highly visible and efficiently emitting internal fluorophore. However, the sensitivity of the GFP reporter system is limited by the lack of amplification, because each GFP molecule produced by the reporter system yields only one fluorophore. It has been calculated that the concentration of natural unmodified GFP molecules should amount to 1 μM in order to match the endogenous autofluorescence of a typical mammalian cell [22]. Mutant (enhanced) GFPs with improved extinction coefficients increase the imaging efficiency by 6–10 times [23], which makes it possible to overcome the limitations associated with cell autofluorescence.

Mammalian tissues have the highest transparency in the so-called “near-infrared (NIR) transparency window” (λ ~ 650–900 nm) [24]. The absorption of light by hemoglobin, water, lipids, and melanin is the lowest in the NIR spectrum region. For this reason, the NIR light has a greater penetrating power than visible light. Moreover, autofluorescence of biological tissues and light scattering are also significantly lower in the NIR region compared to the visible spectrum. To date, fluorescent proteins emitting light in the NIR range are being developed in order to improve the fluorescence intensity in vivo [25]. An example of such proteins is the new NIR fluorescent proteins (iRFPs) developed based on bacterial phytochrome photoreceptors [26]. These proteins provide tissue-specific contrast without the need for any additional substances. Compared to conventional GFP-like red-shifted fluorescent proteins, iRFP670 and iRFP720 show stronger photoacoustic signals at longer wavelengths and can be spectrally distinguished from each other and hemoglobin. Moreover, iRFP670 and iRFP720 do not require oxygen to form chromophores, which gives them an advantage in imaging hypoxic tumors [27, 28].

A number of recombinant oncolytic viruses, including Newcastle disease virus (NDV), vesicular stomatitis virus (VSV), herpes simplex virus type I (HSV-1 or Human alphaherpesvirus 1 according to the new taxonomy of viruses), measles virus (MV), adenovirus (ADV), and vaccinia virus (VACV), encoding fluorescent protein transgenes, have been constructed. These viruses were tested in various tumor models in order to directly assess the effectiveness of viral oncotherapy, and were also used as an additional control to evaluate the effects of other imaging technologies [29 , 30, 31]. One of oncolytic viruses, namely VACV encoding the GFP transgene (GLV-1h68), is currently undergoing phase I and II clinical trials, which use GFP fluorescence to confirm virus localization in superficial tumor sites and biopsy samples from internal tumors (Table) [32].

**Table:** Reporter transgenes of oncolytic viruses

Imaging technique		Reporter transgene	Oncolytic viruses encoding a reporter transgene	Ref.
Optical imaging	Fluorescence imaging	Fluorescent proteins (GFP, eGFP, iRFP)	NDV, MV, HSV-1, ADV, VACV (GLV-1h68), VSV	[14, 29, 55]
	Bioluminescence imaging	Luciferases (FLuc, RLuc, GLuc)	HSV-1, VACV, ADV, MV	[47, 50, 56, 57]
	Photoacoustic imaging	Melanogenic enzymes (Tyr, Tyrp1, Tyrp2)	VACV	[53]
Deep-tissue imaging	SPECT and PET	Enzymes (HSV1-tk)	VSV, ADV, HSV-1	[58 , 59, 60]
	PET	Receptors (SSTR2)	ADV, VACV	[61, 62]
	SPECT and PET	Carrier proteins (NET, NIS)	ADV, VACV, HSV-1, MV	[63 , 64, 65, 66, 67, 68, 69, 70]
	MRI	Melanogenic enzymes (Tyr)	VACV	[53]

A group of Japanese researchers used an oncolytic ADV expressing the GFP transgene (OBP-401 strain) to study a new technology of fluorescence-guided surgery (FGS) for accurate tumor imaging in mice [33]. Surgical resection remains the most effective method for most solid tumors; however, even after radical resection of malignant tumors, relapses often occur, which in some cases may be due to the difficulty of correctly imaging the tumor margin [34, 35]. Pre-injection of a fluorescent oncolytic virus can provide intraoperative real-time fluorescence control and is ideal for an accurate and complete resection of malignant cells. In addition, it allows for further reduction of the resection area due to tumor lysis. Kishimoto et al. used OBP-401-based FGS for a human glioblastoma xenograft in the orthotopic mouse model. The use of a fluorescent oncolytic virus can enable accurate resection of glioblastoma with an indistinct margin by FGS with preservation of brain function and the absence of relapses for more than 120 days. For comparison, 85% of mice with a tumor removed using standard surgical methods had relapses. The OBP-401 strain was also used to study the effectiveness of FGS technology in mouse models of disseminated colon and lung cancer, as well as in soft tissue sarcoma [36].

**Bioluminescence imaging**

Unlike fluorescence, bioluminescence imaging does not require excitation light to emit photons from the fluorophore. Light is produced through substrate oxidation by a bioluminescent reporter enzyme whose gene can be cloned into the genome of an oncolytic virus. The bioluminescence approach features a higher sensitivity (it requires as low as 10-17 M of luciferase) and lower background luminescence compared to fluorescence [37, 38].

Firefly luciferase (Photinus pyralis, FLuc) is the most widely used reporter enzyme for bioluminescence [39]. D-luciferin is used, along with the ATP, Mg^2+^, and O_2_ cofactors, as a FLuc substrate. FLuc catalyzes the formation of the luciferin–ATP complex, whose oxidation leads to production of high-energy oxyluciferin. Oxyluciferin emits photons of the yellow-green spectrum (λ_max_ ~ 560 nm) [40]. Light emission reaches its peak 10–12 min after luciferin injection and gradually decreases over the next 60 min [41]. In addition, ATP-independent luciferases such as sea pansy luciferase (Renilla reniformis, RLuc) [42], marine copepod (Gaussia princeps, GLuc), and click beetle (Pyrophorus plagiophthalamus) luciferases are known [43]. RLuc and GLuc use coelenterazine (CTZ) as a substrate and emit mainly blue light (λmax ~ 460–480 nm), which penetrates tissues worse than the yellow-green light of FLuc [44]. Additional disadvantages of ATP-independent luciferases include their limited distribution, fast kinetics, and higher background noise [37]. New variants of enzymes and substrates with improved bioluminescence are constantly being developed. An example of a new enzyme variant is NanoLuc (λmax ~ 460 nm), whose gene has a shortened coding sequence. This is the only bioluminescent transgene variant for oncolytic viruses with a small genomic capacity, such as adeno-associated virus and other parvoviruses [45]. The use of several types of luciferases allows for simultaneous monitoring of different but related biological events. In particular, the method of labeling tumor cells and an oncolytic virus with various types of luciferases is widely used to determine the antitumor and antimetastatic activities of the virus in in vivo experiments [46].

The first oncolytic virus whose properties were studied by bioluminescence was HSV-1 [47]. Using recombinant HSV-1 variants expressing the FLuc and RLuc transgenes in mouse models, FLuc was shown to provide a more effective monitoring of viral infection than RLuc [48]. Treatment of virus-infected mice with the antiviral drug valaciclovir caused a dose-dependent decrease in the Fluc signal, which was a demonstration of the possibility of quantifying the effectiveness of antiviral therapy in animal models using bioluminescence [48]. In order to obtain more comprehensive quantitative data, bioluminescence is combined with ex vivo imaging of animal organs and a determination of their absolute viral load by real-time PCR [49].

Bioluminescence imaging has been successfully used to determine the effect of combination therapy on mouse tumors using oncolytic VACV together with a blockade of immune checkpoints [50], as well as to monitor replication of oncolytic parvoviruses, adenoviruses, HSV-1, VACV, MV, and VSV within a tumor in mouse models (Table) [37].

**Photoacoustic imaging**

Photoacoustic imaging, or optoacoustic imaging, is a recently developed imaging modality that uses the photoacoustic effect produced by irradiation of the target tissue with a short-pulsed laser. The tissue, depending on its physical properties, absorbs different amounts of light, which causes molecular vibration and thermoelastic expansion [24, 51]. Acoustic waves resulting from this process are less scattered than photons passing through tissue, which greatly increases image resolution [24]. Sometimes contrast agents are used to increase the molecular specificity of photoacoustic imaging [52].

To date, photoacoustic imaging experiments have been conducted using only one oncolytic virus, VACV, which expresses the key genes for melanin production: the tyrosinase (Tyr) gene and genes encoding the Tyr-related proteins 1 (Tyrp1) and 2 (Tyrp2) (Table) [53]. Melanin is an ideal contrast agent for photoacoustic imaging, while expression and accumulation of melanin in tumors make it possible to use photoacoustic imaging in oncotherapy experiments in animal models [53]. However, high concentrations of melanin inhibit viral replication; therefore, in order to reduce this inhibitory effect, an inducible system regulated by doxycycline is used to express Tyr group transgenes [54]. The complexity of choosing transgenes for photoacoustic imaging still limits the use of this technique in oncolytic virotherapy, despite its high resolution.

## DEEP-TISSUE IMAGING

The transition from in vitro and preclinical studies to clinical trials requires appropriate translational animal models to adequately evaluate safety and efficacy. For this purpose, it is necessary to use large animals that are physiologically close to humans, such as dogs, pigs, and primates, since they allow one to better predict the clinical outcome of a therapy than when using small animals like mice and rats [71]. Optical imaging modalities used in small animals are not applicable to large animals because visible light cannot penetrate the tissues of large animals [18, 72]. For this, deep-tissue imaging techniques such as single-photon emission computed tomography (SPECT), positron emission tomography (PET), and magnetic resonance imaging (MRI) are required (Fig. 1) [73].

Imaging by nuclear medicine techniques (PET and SPECT) is based on a recognition and localization of the gamma rays emitted during the decay of a radioactive tracer introduced into the patient’s body and accumulated specifically in various organs and tissues [74]. Specialists can draw a conclusion about the state of health of the organ under study and its metabolic activity based on how the cell reacts to the introduction of a radioactive drug, how this drug accumulates, and how it is being excreted. The spatiotemporal pattern of radiopharmaceutical distribution provides an idea of the organ’s shape, size, and position, as well as the presence of pathological lesions in it [16].

SPECT uses radiopharmaceuticals labeled with radioisotopes, whose nuclei emit only one gamma quantum (photon) during each radioactive decay act. PET utilizes radioisotopes emitting positrons, which, in turn, when annihilated with an electron, yield two gamma quanta moving in different directions along the same line; this increases PET sensitivity compared to that of SPECT [75]. SPECT and PET are often combined with computed tomography (CT) for co-registration of anatomical and functional images. A large set of detectors located around the object under study during PET and computer processing of the signals received from them make it possible to perform a more accurate three-dimensional reconstruction of the radionuclide distribution in the scanned object compared to SPECT [16].

MRI uses strong magnetic fields and radio waves to excite the nuclear–spin energy transition of hydrogen molecules. Hydrogen nuclei are present in large quantities in the human body in the composition of water and other substances. The rate of relaxation of nuclear hydrogen atoms from their excited state depends on tissue density, and this difference makes it possible to obtain sufficiently high-resolution images [76]. The disadvantages of MRI include the high cost of the devices and the long time required to obtain images (15–90 min).

Most oncolytic virus transgenes utilized in deep-tissue imaging can be studied using various techniques depending on the substrate. These transgenes encode HSV1 thymidine kinase (HSV1-tk), somatostatin receptor 2 (hSSRT2), enzymes catalyzing melanin synthesis (Tyr), and such transporter proteins as the human norepinephrine transporter (hNET) and the sodium/iodide symporter (NIS) [14, 63, 73].

**Enzyme reporter transgenes**

One of the first reporter genes proposed for non-invasive radionuclide imaging was the HSV1-tk gene. Its product, thymidine kinase, phosphorylates thymidine to thymidine 5’-monophosphate. Unlike mammalian thymidine kinase type 1, which has a high affinity mainly for thymidine, HSV1-tk exhibits specificity to various nucleosides. For example, HSV1-tk can phosphorylate both pyrimidine analogs (5-iodo-(2’-deoxy-2-fluoro-β-D-arabinofuranosyl)uracil, FIAU; 2’-deoxy-2-fluoro-arabinofuranosyl-5-ethyluracil, FEAU; (E)-5-(2-bromovinyl)-2’-deoxyuridine, BVDU) and acycloguanosine derivatives: acyclovir (ACV), ganciclovir (GCV), and 9-[4-fluoro-3-(hydroxymethyl) butyl]guanine (FHBG). HSV1-tk specifically interacts with radioactively labeled pyrimidine analogs, converting them into a metabolite incapable of leaving the cell, resulting in accumulation of the transformed radioactive substrate in the cells expressing HSV1-tk [77, 78]. Since HSV1-tk is one of the first well-characterized reporter genes, it has a wide range of substrates for PET and SPECT, as well as mutant forms such as HSV1-sr39tk, which have increased activity in vivo [73].

The HSV1-tk transgene was used to assess the biodistribution of recombinant oncolytic VSV in rat models of hepatocellular carcinoma [58]. HSV1-tk-expressing ADV capable of displaying virus localization using PET scanning was also obtained (Table) [59]. The first clinical studies were performed to evaluate the possibility of using the recombinant oncolytic HSV1-tk strain HSV1716 as a reporter transgene to monitor viral replication during treatment of glioma patients [60]. However, increased substrate (^123^I-FIAU) accumulation in tumor cells was not registered by SPECT, which may be due to both insufficient virus replication and the low sensitivity of the method. In addition, HSV1-tk, as a foreign protein, can elicit an immune response, making it unsuitable for long-term imaging, which is important for gene therapy.

**Receptor proteins as reporter transgenes**

SSTR2, one of the receptors for the peptide hormone somatostatin, is expressed on neuroendocrine and other cells, where it is involved in neurotransmission, hormone secretion, and cell proliferation [3, 72]. The human hSSTR2 protein was used for SPECT imaging with indium-111-labeled synthetic peptide substrates such as octreotide, pentetreotide, and lanreotide, as well as PET imaging with gallium-68-labeled peptides [79]. Researchers integrated the SSTR2 transgene into the genomes of the oncolytic viruses ADV and VACV and demonstrated the possibility of long-term monitoring of the localization and persistence of these viruses in the syngeneic mouse models of several cancers [62, 73]. The expression of the SSTR2 transgene in the adeno-associated virus makes it possible to obtain PET images even six months after the end of therapy [61]. The disadvantages of SSTR2 include its endogenous expression, which can reduce the diagnostic performance, and the fact that each receptor can bind only one radiolabeled ligand, making signal amplification impossible and, thereby, limiting the imaging sensitivity [3, 18].

**Contrast agents as transgenes**

The genes of melanogenesis, such as the tyrosinase gene Tyr, which is also used in photoacoustic imaging, can be utilized as reporter genes for MRI imaging [73, 80]. Tyr-induced melanin production enhances the chelation of metal ions, resulting in a significant improvement in MRI contrast. Because the contrast agent is produced directly in the transduced cells, imaging becomes possible without the use of an exogenous contrast agent.

The use of a recombinant VACV strain expressing melanin overproduction transgenes made it possible to carry out MRI imaging of the tumor and metastases in the xenograft model of human metastatic A549 lung cancer cells inoculated in immunodeficient mice. This VACV strain has also been successfully used for photoacoustic imaging (see section “Photoacoustic imaging”) [53].

Melanin is present in all the kingdoms of living organisms. Therefore, melanin synthesis can possibly be used as a diagnostic/theranostic marker for most known species, including humans. [14, 81].

**Carrier proteins as reporter transgenes**

The Na^+^/Cl–-dependent membrane protein NET transports norepinephrine (NE), epinephrine, dopamine, and other structurally related compounds into the cell. Most cells of neuroblastoma (the most common extracranial solid tumor in children, which accounts for 15% of the deaths among all childhood cancers) express NET on their membrane [82]. Meta-iodobenzylguanidine (MIBG), also known as iobenguane, is a structural analog of NE, a natural NET substrate. MIBG was first adapted for the imaging of the adrenal medulla by scintigraphy in the 1980s [83]. Radioactively labeled MIBG (123I-MIBG) can be effectively used for neuroblastoma imaging in the whole body. Currently, gamma scanning with 123I-MIBG is considered to be the preferred method of detecting primary tumors and identifying metastatic neuroblastoma cells [84].

The human NET transgene (hNET) was introduced into the genomes of oncolytic VACV, ADV, and herpes viruses and used for the nuclear imaging of not only neuroendocrine, but also other human cancers in immunodeficient mouse models [64 , 65, 66]. However, the use of exogenous NET for tumor imaging remains extremely limited, which is probably due to the existence of other reporter genes that are more accessible to radioactive tracers and have better expression profiles [85].

Sodium/iodide symporter (NIS) is a transmembrane glycoprotein that mediates iodide uptake and accumulation for organification of thyroid hormones; it plays a central role in the metabolism of thyroid hormones and is also expressed in other tissues, including the salivary gland, gastric mucosa, and the mammary gland [86]. Thanks to its ability to accumulate iodide, NIS has been used to detect and treat thyroid diseases, demonstrating the clinical versatility and practicality of the NIS-mediated iodide uptake, for more than 75 years [87]. Vector delivery of NIS allows for iodide accumulation in the tissues of other organs, where NIS is not normally expressed. NIS is responsible for the intracellular transportation of various types of gamma-emitting radioisotopes that are readily available and approved for use in humans. These radioisotopes include radioactive iodine (123I, 124I, 125I, and 131I), technetium in the form of anionic pertechnetate (99mTcO4-), and perrhenate (^186, 188^Re), which are suitable for non-invasive SPECT and PET imaging [14]. Ectopic expression of NIS ensures the accumulation of radioactive iodide either at a comparable or higher level as that of thyroid cells without affecting the main biochemical processes taking place in the cell [88]. This expands the scope of radiotherapy and NIS imaging use beyond the thyroid gland.

The use of NIS has potential advantages over other reporter gene systems. Unlike receptor-based reporters with stoichiometric linkages such as hSSTR2 (in which the receptor can only bind one radiolabeled ligand, preventing signal amplification and limiting imaging sensitivity), transporters such as NIS provide signal amplification via the intracellular transport-mediated accumulation of the substrate, thereby increasing detection sensitivity [73, 89]. NIS imaging was also proved to be more sensitive and longer lasting compared to HSV1-tk imaging [90]. NIS can show cell viability, since the accumulation effect of NIS is lost during cell apoptosis, while enzymes and receptors can still retain their functional activity [63]. NIS is found in all vertebrates, which makes it possible to use the species-specific NIS transgene in the vast majority of model systems [91, 92].

NIS not only has the advantages described; it is also the most abundant human reporter transgene. Many NIS-expressing recombinant viruses have been developed. NIS-encoding non-replicating ADV, which was studied in the xenografts of various human cancers, including cervical cancer, breast cancer, and prostate cancer (PCa), as well as in immunodeficient mouse models, was the first NIS-expressing virus obtained [67]. Shortly thereafter, a high-resolution SPECT image of canine prostate cancer tissue was obtained using replication-competent ADV expressing the NIS symporter (Ad5-yCD/mutTK[SR^39^]rep-hNIS) [93]. Phase 1 clinical trials of Ad5-yCD/mutTK[SR^39^] rep-hNIS were also conducted in a group of men with clinically localized PCa, which proved the possibility and safety of non-invasive SPECT imaging for monitoring the effectiveness of ADV-mediated gene therapy in humans [94]. The replication and distribution of recombinant oncolytic VSV (VSVd51-NIS) was monitored in mice transplanted with subcutaneous 5TGM1 myeloma by serial 123I-γ-scintigraphy after systemic and intratumoral administration [68]. Clinical trials showed the efficacy of using another recombinant VSV strain, VSV-IFNβ-NIS, to image metastatic colorectal and pancreatic cancers [95]. VACV expressing the hNIS transgene successfully inhibited the growth of several cancers in preclinical models, including pancreatic cancer, triple-negative breast cancer, gastric cancer, and malignant pleural mesothelioma [96, 97, 98]. Recombinant MV (Edmonston strain) expressing hNIS has undergone and is currently undergoing the largest number of phase 1 and 2 clinical trials in various cancers, including ovarian cancer (NCT02068794), head and neck squamous cell carcinoma and breast cancers (NCT01846091), malignant peripheral nerve sheath tumor (NCT02700230), multiple myeloma (NCT00450814), and urothelial carcinoma (NCT02364713) [3, 69, 70].

A major limitation of NIS imaging is the accumulation of radioisotopes in such NIS-expressing non-target tissues as thyroid and salivary glands and stomach. If the transduced tissue is adjacent to endogenous NIS-expressing tissues, then interpretation and quantification of NIS signals are technically difficult. Several studies have explored ways to improve NIS expression and block endogenous NIS expression in order to tackle these issues [99, 100].

## CONCLUSION

As we can conclude from the presented data, the introduction of reporter transgenes into the genome of oncolytic viruses is a promising tool for a non-invasive molecular imaging of tumor tissue to assess tumor localization, size, and the effectiveness of its treatment. The choice of a reporter transgene depends on the imaging techniques used, which can be divided into two main categories: optical imaging and deep-tissue imaging.

Optical imaging techniques are amiable due to their short acquisition times, low cost, high throughput capacity, lack of toxicity in animal models, multispectral imaging capabilities, and ease of use compared to the radioisotopes required for deep-tissue imaging. These properties make optical imaging an extremely popular approach for in vitro and preclinical studies in small animals.

Transgenes of fluorescent proteins (fluorescence imaging), luciferase (bioluminescence imaging), and Tyr enzymes (photoacoustic imaging) are used for optical imaging of the antitumor properties of oncolytic viruses.

The limitations of optical imaging techniques include a shallow penetration depth, the absorption and scattering of the excitation and/or emitted light, especially in deep tissues; and the presence of cell autofluorescence, including that of dead cells, which is of particular importance when using oncolytic viruses that lyse tumor cells. Although the attenuation of the light flux and autofluorescence can be minimized within the infrared “window,” optical imaging methods have a low spatial resolution and limited sensitivity. These problems, as well as the risk of developing immune responses to the foreign reporter proteins encoded by the transgenes of oncolytic viruses, prevent the adaptation of optical imaging modality for large animal and human models and, therefore, their use in the clinic.

Deep-tissue imaging techniques (SPECT, PET, and MRI) have the most translational potential, thus making it possible to study animals of any size, including humans. The same reporter transgenes of oncolytic viruses can be used for deep-tissue imaging by different methods depending on the contrast agent used. Melanin is an ideal MRI contrast agent; therefore, melanin-producing Tyr genes are used as transgenes of oncolytic viruses. However, melanin at high concentrations inhibits viral replication, which significantly limits the use of these imaging modalities in tumor virotherapy. Nuclear imaging methods (SPECT and PET) use radioactive isotopes as a substrate; a number of oncolytic virus transgenes have been developed for accumulation of these isotopes in tumor cells. These transgenes encode enzymes (HSV1-tk and its modifications), receptors (hSSRT2), as well as carrier proteins such as hNET and NIS.

One of the oldest and most effective reporter genes, NIS, is used for molecular imaging and targeted radionuclide therapy. NIS is found in all vertebrates, which makes it possible to use the species-specific NIS transgene in the vast majority of model systems. Not only preclinical, but also clinical studies confirm that NIS, expressed in oncolytic viruses, can be used to accurately determine tumor localization and response to therapy, as well as detect metastases using deep-tissue nuclear imaging.

## Abbreviations

GFP: green fluorescent protein
NIR: near-infrared
ADV: adenovirus
HSV-1: herpes simplex virus 1
MV: measles virus
NDV: Newcastle disease virus
NIS: sodium/iodide symporter
VACV: vaccinia virus
VSV: vesicular stomatitis virus
FGS: fluorescence-guided surgery
CT: computed tomography
MRI: magnetic resonance imaging
SPECT: single-photon emission computed tomography
PET: positron emission tomography
Tyr: tyrosinase
PCa: prostate cancer
